# Battery-Less Industrial Wireless Monitoring and Control System for Improved Operational Efficiency

**DOI:** 10.3390/s23052517

**Published:** 2023-02-24

**Authors:** Eduardo Hidalgo-Fort, Juan Antonio Gómez-Galán, Ramón González-Carvajal, Pedro Sánchez-Cárdenas, Carlos Clemente-Maya

**Affiliations:** 1 Department of Electronic Engineering, University of Seville, 41092 Seville, Spain; 2 Department of Electronic Engineering, Computers, and Automation, University of Huelva, 21007 Huelva, Spain; 3 Atlantic Copper S.L.U. Company, Avda. Francisco Montenegro, s/n, 21001 Huelva, Spain

**Keywords:** industrial wireless monitoring, wireless sensor network, battery-less, LoRaWAN, energy harvesting

## Abstract

An industrial wireless monitoring and control system, capable of supporting energy-harvesting devices through smart sensing and network management, designed for improving electro-refinery performance by applying predictive maintenance, is presented. The system is self-powered from bus bars, and features wireless communication and easy-to-access information and alarms. With cell voltage and electrolyte temperature measurements, the system enables real-time cell performance discovery and early reaction to critical production or quality disturbances such as short-circuiting, flow blockages, or electrolyte temperature excursions. Field validation shows an increase in operational performance of 30% (reaching 97%) in the detection of short circuits, which, thanks to a neural network deployed, are detected, on average, 10.5 h earlier compared to the traditional methodology. The developed system is a sustainable IoT solution, being easy to maintain after its deployment, and providing benefits of improved control and operation, increased current efficiency, and decreased maintenance costs.

## 1. Introduction

Advanced monitoring systems data have continued to increase remarkably over the last years in the industry, where data integration and fast-track analysis with a focus on serving daily operations and decision making is one of the key opportunities in improving operational efficiency and competitiveness [1,2,3]. Traditionally, wired communications have been employed to transmit information since they offer a good level of reliability and security. Nevertheless, they also have constraints to supervise areas of the factory that are difficult to access, the corrosion can affect their performance, and the topology of the wired network presents low flexibility. The expansion in the last years of wireless sensor networks (WSN) to a number of new applications is thanks to the spread of smart sensors and the development of communication protocols. The industries are also moving toward complete digitalization, and WSNs help address the aforementioned issues, replacing their wired counterparts [4,5,6]. The industry has already started to benefit from these promising wireless systems in terms of simple deployment, low cost, reduced maintenance tasks, spatial flexibility, scalability, and robustness. However, the industrial wireless automation must face several challenges because the reliability of the transmission must be assured in an ever-complex environment with areas of imperfect radio coverage, or where other networks can also coexist [7,8]. Hence, a careful design and a suitable deployment is necessary to avoid possible interferences and an unacceptable number of collisions between transmissions in situations of high traffic.

In general, energy consumption has not been a major concern for industrial wireless systems. The importance of energy consumption for industrial wireless networks has increased only recently, as wireless industrial technologies have commenced to merge with internet technologies. To fulfil this vision, the Internet of Things (IoT) allows for a huge amount of information to be collected from a large number of smart sensors, with low sampling rates, to collaborate with each other, providing an intelligent service [9,10]. Continuous advances in microelectronics have made possible low-cost and more powerful sensors equipped with sensing, computation, and wireless communication to perform multiple tasks for handling data, such as collection, processing, transmission, storing, and analysis. These devices introduced as part of the IoT have design constraints that are dependent on both the application and the monitored environment [11]. A common concern of IoT devices is the need for a long lifetime. Currently, batteries are the dominant power source for IoT devices, along with (but not necessarily) energy harvesting solutions, and thus, the reduction in the power consumption of the circuits is mandatory [12,13,14]. Batteries, even when rechargeable, have a lifetime that is limited by the number of charge cycles. The periodic replacement of node batteries is an unaffordable task as the number of devices grows.

Promising alternatives for a sustainable IoT are the emerging battery-less devices, which harvest energy from the environment and store it in supercapacitors [15]. Although batteries provide higher storage capacity, supercapacitors offer advantages in terms of size, lifetime, and temporal pulse capability, lasting even more than a decade. Nevertheless, the power budget of these self-powered systems must be constrained based on the environment in which they are deployed, since they must provide reliability, especially under poor, dynamic, or unpredictable harvesting conditions, which can cause power failures. Usually, this fact forces the communication of the harvester-powered device to be conservative. Given the nearly maintenance-free operation of battery-less devices, they are more suitable for deployment in hard-to-reach locations.

Significant efforts towards the reduction in both energy consumption and device size, as well as advancements in the energy-supplying circuits, are the leading aspects for the development of sustainable IoT technologies, together with the way these devices communicate with each other. IEEE 802.15.4, ZigBee, Bluetooth, DASH7, and WirelessHart have been deployed for different applications, and they were the first technologies that implemented low-rate wireless personal area networks (LR-WPAN) [16,17,18]. Subsequently, other wireless radio technologies with low power consumption and a wide range coverage (LPWAN) have been developed, such as Sigfox, LoRa/LoRaWAN, NB-IoT, and LTE-M, using the sub-1GHz bands [19,20,21]. LPWANs extend the coverage with low power consumption, optimizing the modulation scheme and simplifying the network topology. In this work, we consider IoT devices using the LoRaWAN technology. The LoRaWAN communication protocol is based on the LoRa technology, which was one of the first long-range technologies to become commercially available, and the most frequently applied [22,23,24]. It allows a system to provide network service and enable devices to wirelessly transfer data over long distances to remote gateways.

Despite the attempts to use modern monitoring and control techniques in the industry to overcome some of the aforementioned drawbacks, in some fields, their level of use has been extremely low. This is the case of the target application, related to the copper production process in electro-refineries, due to the huge and hostile work area to be instrumented, the difficult relations between chemical, physical, and electrical process parameters, and a strongly stabilized manual supervision [25,26,27]. This paper aims to develop a wireless remote monitoring and control system of electrolytic cells to improve the performance, the operability, and the maintenance of the copper electro-refining process in real time, which increases the performance of short-circuit detection and prevention with respect to traditional methodologies. The system is based on a wireless sensor network and includes all the components required to provide real-time monitoring while yielding increased operational efficiency. The proper performance of the whole system relies on the operation of the wireless sensor modules attached to the electrolytic cells in order to gather the relevant information and send it to the centralized node, where it is processed by the deployed neural network. Unlike the rest of the state-of-the-art solutions, the system is powered by the cell bus bar voltage and provides real-time measurements for voltage and electrolytic temperature. This energy-harvesting approach uses a supercapacitor as an energy storage technique, providing a sustainable WSN. The sensing nodes are placed close to the bus bar, minimizing cabling, and simplifying the installation and the maintenance. The designed power supply system can measure cell voltage data with high accuracy, in addition to powering the system. Moreover, the power consumption of the wireless sensor network has been optimized both at a hardware and software level, which allows for a time-limited autonomous operation, even during the copper harvesting phase, where the power supply is absent. Furthermore, the secure wireless communication ensures an efficient and real-time detection of disturbances associated to the process. All this makes the nodes more suitable for deployment in locations hostile for electronics, such as the application under consideration (high corrosion levels, moderate temperature, large magnetic fields, and liquid spills).

The paper is organized as follows: the battery-less industrial wireless system overview is presented as an entry point in Section 2 before moving forward to the architecture definition, design, physical implementation in Section 3. Section 4 describes the firmware and the graphical user interface. Section 5 deals with the measurement results. Finally, conclusions are explained in Section 6.

## 2. System Overview

Copper production involves several phases culminating in the electro-refining process, which obtains cathodes with a purity greater than 99.99% from copper anodes with a minimum purity of 99.5% [28]. The impure anodes and cathodes (stainless steel sheets at first) are interleaved and placed into cells filled with electrolyte (sulfuric acidic solution). The copper ions are released from the anodes to the cathodes due to the several tens of thousands of amps of DC current applied to the bars of the electrolytic cells [29]. It is a cyclic process in which several collections of cathodes are pulled out for each anode. The copper growth on cathodes is influenced by many parameters, such as current density, electrolyte temperature, anode composition, anode–cathode spacing, electrolyte flow, and dosage and concentration of additives. One of the persistent problems in copper electro-refining operations that degrades the quality of the high-purity cathodes is the growth of nodules on the copper cathodes, caused mainly by particles contained in the electrolyte [30,31,32]. This fact causes a large number of short circuits between anodes and cathodes that require great attention in manual maintenance tasks, leading to a decrease in production capacity [33,34]. Furthermore, the refining plant is very extensive, with tens of hundreds of cells and thousands of cathodes and anodes within a harsh environment. Thus, the lack of online measurement systems and the low dynamic of the process disturbances complicate the detection of short circuits.

Short circuits between electrodes raise the temperature and drastically decrease the current efficiency. Currently, the detection of short circuits is performed by walking on top of the cells of the plant with conventional instruments such as Gauss meters (to measure the intensity of the magnetic field, which is proportional to the cathode current) and thermal cameras [35]. This is a very time-consuming technique requiring a large number of hours for the large area to be checked thoroughly, leading to a relatively high misdetection rate, and even entailing certain risks for the operators. The infrared image method has received attention for the electrode state detection [34,36]. However, the electrolytic cells are generally covered to avoid electrolyte evaporation, making it difficult to take the infrared image. In addition, this method does not enable preventive maintenance since the electrode must be very hot (i.e., the short circuit occurred for a long time) for proper detection, increasing the current losses. Other methods are based on the short-circuit current measurement. Hall sensors have to be used to estimate the current by collecting the magnetic induction intensity of the cathode, but the small distance between the sensor and the cell bus bar leads to coupling, which hinders the current estimation at each cathode [37]. Other recent research works have reported the study of measuring the anode current or the cathode current using optical fiber current sensors [33,38], by analyzing the change in the current slope and the deviation from the normal working current.

**Figure 1:**
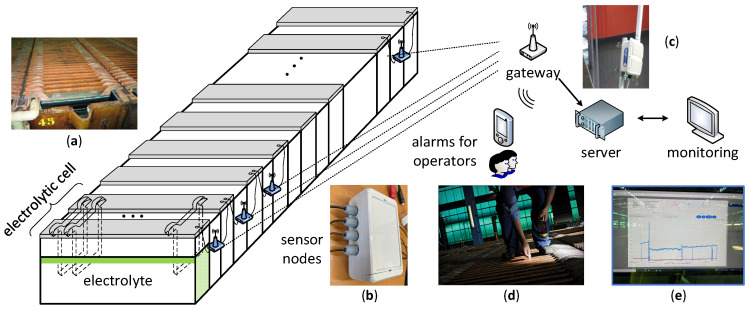
Proposed general system structure. (**a**) Image of the anodes and cathodes of an electrolytic cell. (**b**) Sensor–actuator node. (**c**) Gateway. (**d**) Operator removing a short circuit. (**e**) Monitoring of cell parameters trend with associated process disturbances.

This work addresses the wireless automation of the copper refining process to overcome some of the aforementioned drawbacks. Figure 1 shows the general structure of the innovative refinery monitoring and control system. It is a powerful data collection system based on robust wireless communication and powered by the cell bus bar voltage. The proposed industrial WSN architecture comprises three main subsystems: sensor devices, the base station or gateway, and the information management system. Despite being a common structure, each of the elements requires specific considerations for achieving the efficiency demanded by industrial applications.

The proposed system provides real-time status for each cell to which it is installed. Figure 1a shows a photograph of the anodes and cathodes of an electrolytic cell. The status is based on cell voltage and electrolyte temperature measurements managed by advanced, self-powered, and wireless sensor–actuator nodes placed at the cells, as shown in Figure 1b. The gateway (Figure 1c) is responsible for the self-organizing wireless network management and for establishing a communication channel between the sensing nodes and the central server. The cell status is calculated continuously with a neural network by the management system, which is located in the central server. The alarms generated by the system are immediately sent to the operators, as shown in Figure 1d. The identified short circuits are removed by manually hitting the nodules (shorts) growing between anodes and cathodes.

On the other hand, the copper refining process requires keeping up a continuous electrolyte flow circulating through the cells to remove impurities dissolving from the anodes and to maintain the desired temperature (about 65 °C) and additive levels [39]. For this reason, the wireless system must also measure the electrolyte temperature, since a decrease indicates poor flow levels. Finally, web and mobile applications that implement a graphical user interface have been developed, allowing the user to configure and manage all devices of the wireless system, as well as monitoring and receiving alarm warnings. Figure 1e shows as an example a cell voltage trend with associated process disturbances. The proposed solution allows improving the efficiency of the process in terms of current consumption, energy, cathode copper quality, time efficiency of the refinery, and maintenance costs.

## 3. Hardware Description

A self-sustainable wireless sensor network which implements an energy-harvesting mechanism has been achieved, since it is difficult to change or recharge batteries after the deployment. This strategy has been combined with low-power electronic designs at a hardware level and with operation techniques in the firmware, resulting in efficient power management. The hardware of the system was implemented using low-cost commercial off-the-shelf items. The hardware of the IoT monitoring and control system comprises a wireless sensor and actuator network, which implements a LoRaWAN star topology for communication between the LoRa gateway, which communicates with the central server through an ethernet connection and the wireless nodes. The tests carried out guarantee a good level of coverage throughout the plant, as well as an adequate channel occupation, as detailed in Section 3.4. On the other hand, the implementation of a star network minimizes the probability of failure in the transmission of information, because the information only makes one hop and the transmission of a node does not depend on the state of other nodes which, being supplied from the electrolytic cells, can be turned off if their cells are in the process of harvesting.

**Figure 2:**
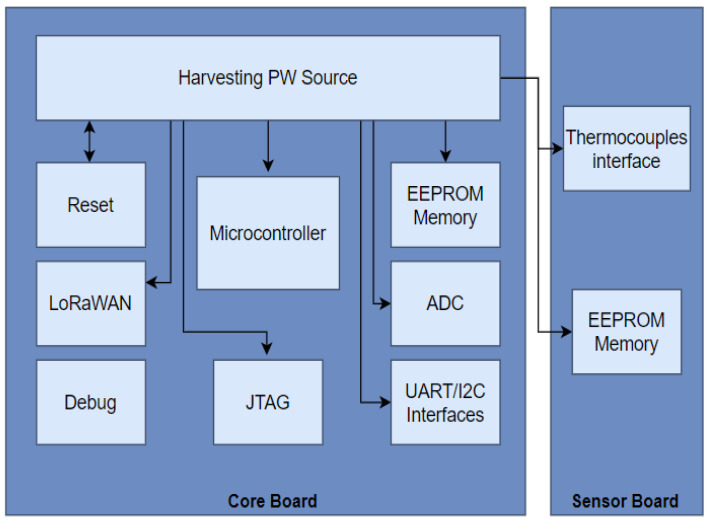
Block diagram of the hardware design.

The hardware design consists of three main parts: (1) a power supply system, (2) a microcontroller and peripherals, including sensor interfaces, and (3) radio technology. Figure 2 shows the general block diagram of the hardware used for the sensor–actuator prototype node, consisting of two electronics-printed circuit boards: core and sensor board (SB). The system was optimized to require the deployment of as few nodes as possible. Thus, each sensor–actuator node is designed to manage up to four cells, which involves a trade-off between simplicity of the hardware design, power consumption, and duty cycle of the communication channel.

### 3.1. Microcontroller and Peripherals

The selection of a microprocessor becomes important in power-aware design. The core board is governed by a 32-bit STM32L152RET6 microprocessor from ST Microelectronics with ARM Cortex-M3 architecture, which operates in a range of 1.65 V to 3.6 V at 8 MHz with an external oscillator. A second ABS25 crystal oscillator provides a precise 32.768 kHz digital clock signal with an accuracy of ±20 ppm for RTC (real-time clock). The microcontroller is well suited for this application because of its low-power operating current, which is 195 µA/MHz in run mode and 1.11 µA for standby current in sleep mode, including the RTC. The sensor interface is designed to be compatible with UART and I2C for possible future needs, and also includes an external JTAG connector and a 19 × 2 pin connector for SB connection. Five analog inputs are reserved for the bus bar cable connections, to measure the voltage of four cells. A supercapacitor voltage monitor is included through a high-impedance voltage divider coupled to one of the available analog-to-digital inputs of the microcontroller. A 24LC1025 EEPROM memory from Microchip completes the interfaces.

The sensor board includes four k-type thermocouples for temperature probe connections which are protected against the electrolyte by a Teflon capsule, and a 24LC128 EEPROM memory which identifies the sensor board. The measurements of the thermocouples are conditioned by means of the instrumentation amplifier AD8495 from Analog Devices, which produces a high-level (5 mV/°C) output directly from the thermocouple signal by combining an ice point reference with a precalibrated amplifier.

### 3.2. Power Supply System

The main challenge to design the power supply system is to conciliate an accurate measurement of the low cell voltage, which in turn is used to power the entire system. Two separated paths are required. While one is reserved for the sensor interface, which allows for the connection of the cell voltages to an analog-to-digital converter input of the microcontroller, the other powers the microcontroller and all other components. The measurement path includes several Schottky diodes BAT54S to protect the system from high peak input voltages, which can take place when a group of cells is turned off to pull out the pure cathode copper plates. The power supply path includes an LT3105, which is a high-efficiency step-up DC/DC converter from Linear Technology that can operate from input voltages as low as 225 mV (as required for the low cell voltages) and provides an output voltage of 4.5 V to charge a supercapacitor of 1 F. Next, a low dropout voltage regulator MCP1700 from Microchip provides 3.3 V to power all components, delivering up to 250 mA of current while consuming only 1.6 μA of quiescent current. This high current is required for the short periods of wireless data transmission, which last only 10 ms. The power supply path is protected by a PTC resettable fuse MF-R050 from Bourns, a Schottky diode MBRS540T3, and two CS3D diodes.

### 3.3. Radio Frequency Part

The wireless communication is realized with the HTCC AM01 module, which is a fully integrated LoRaWAN-compatible transceiver module. It includes the SX1262 transceiver from Semtech and an ARM Cortex-M0 processor. The HTCC AM01 module also provides different digital interfaces, such as, UART, SPI, and I2C, and only consumes a current of 3.5 µA in deep sleep mode. The module can be fully controlled through the UART interface with AT commands. This module transmits data received from the UART interface to a LoRaWAN gateway via wireless communication at 868 MHz. In order to maximize the sleep mode time, data from the gateway can be received.

**Figure 3:**
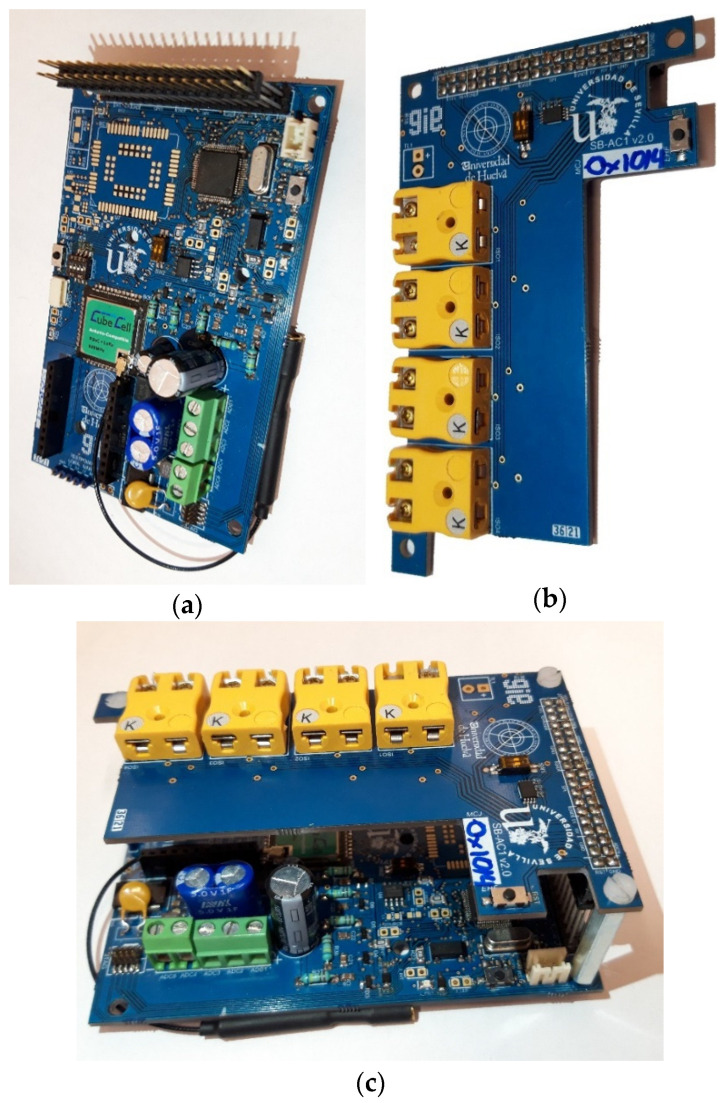
Hardware design. (**a**) Core board. (**b**) Sensor board. (**c**) Assembly configuration.

Figure 3a,b show the details of the hardware implementation of the two boards of the wireless data acquisition node, and Figure 3c shows the assembly configuration. A careful design layout was required to reduce the noise, isolating the signal conditioning and A/D conversion, using large ground planes to avoid interferences from the wireless communication modules, although the integrated antenna connector of the HTCC AM01 module is used. Moreover, the antenna pad is surrounded by pathways connected to ground in order to avoid external radiation that could interfere with its proper operation.

**Figure 4:**
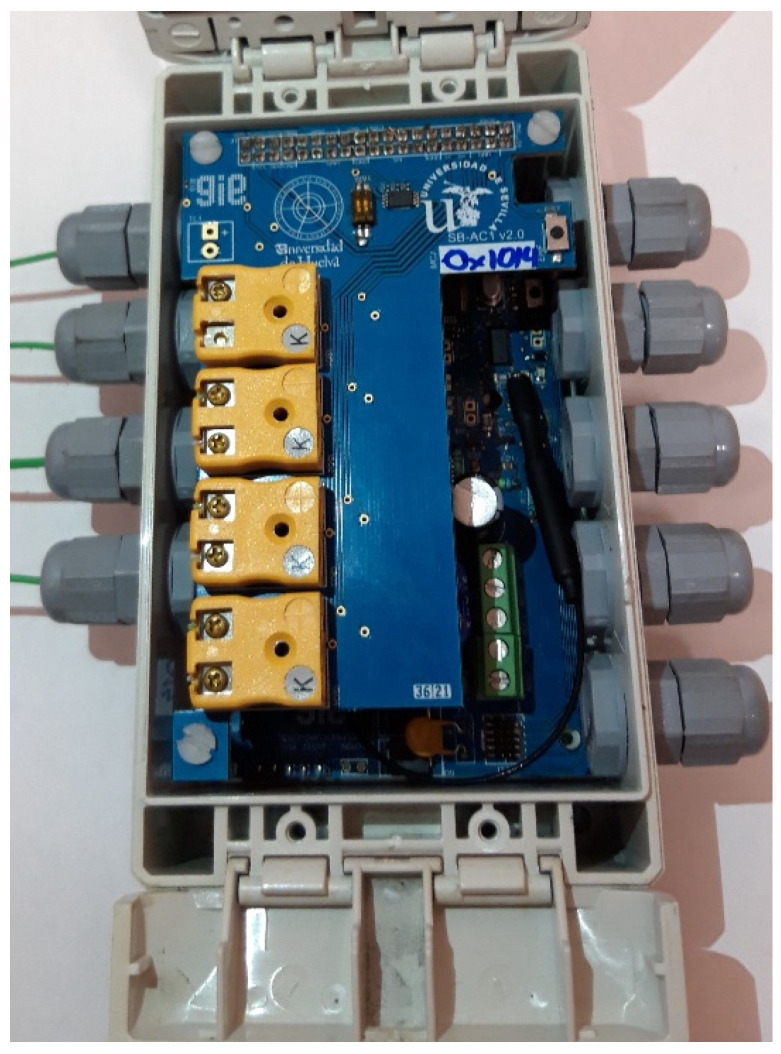
Packaging design.

The node is encapsulated in a waterproof polycarbonate container (151 × 80 × 60 mm) with high robustness and an IP68 protection rating. Cable glands are used as sealing and termination devices to ensure that the characteristics of the enclosure which the cable enters can be maintained adequately. Figure 4 shows the two boards installed inside the open enclosure.

### 3.4. Gateway

The sensor–actuator nodes have been configured as end devices to deploy a point-to-point network topology based on a base station or gateway, to avoid sending the information through operated or public networks and, therefore, guarantee its integrity. The model MTCDTIP-L4E1-266A-868 from Multitech was chosen as the gateway, and it completes the hardware infrastructure of the system. It is a flexible solution that provides durable, low-power, wide-area network connectivity in support of IoT LoRaWAN applications in any environment. Moreover, it is highly scalable and comes with IP67 protection capable of resisting the harshest environmental factors including moisture, dust, rain, and extreme heat.

The wireless communication network was dimensioned taking into account the number of messages to send, and that the electrolysis plant consists of 1204 cells. Thus, the duty cycle (DC) is a key parameter, i.e., the time that the system occupies the communications channel. In LoRaWAN, this parameter must not exceed 1%, a value established by the ETSI (European Telecommunications Standards Institute) for the frequency sub-bands G (863.0–868.0 MHz) and G1 (868.0–868.6 MHz). That is, the system can occupy the channel 36 s every hour. This restriction forced us to design the network based on that limit. It can be calculated as follows: *DC* = (*no of nodes*·*ToA*·100)/(*channels*·*T*), where *T* is the time between the sending of messages, *ToA* (time-on-air) is the time that the message remains in the air, and *channels* is equal to 8 in LoRaWAN. Following the recommendations of [40], *ToA* was calculated through the parameters spreading factor (*SF*), bandwidth (*BW*), code rate (*CR*), and payload. Considering that no LoRaWAN Header or data rate optimization were used, for a payload of 50 bytes, an *SF* of 7 (maximum transfer speed), a *BW* of 125 kHz, and a *CR* of 1 yielded a *ToA* of 97.54 ms, which is equivalent to a maximum of one message every 10 s. To reduce the duty cycle below 1%, and considering that LoRaWAN can support up to eight channels, it we decided that each node would take four cells (301 nodes) and the data would be sent every 8 min, resulting in a DC of 0.76%.

## 4. Firmware/Software Details

### 4.1. Sensor Devices

**Figure 5:**
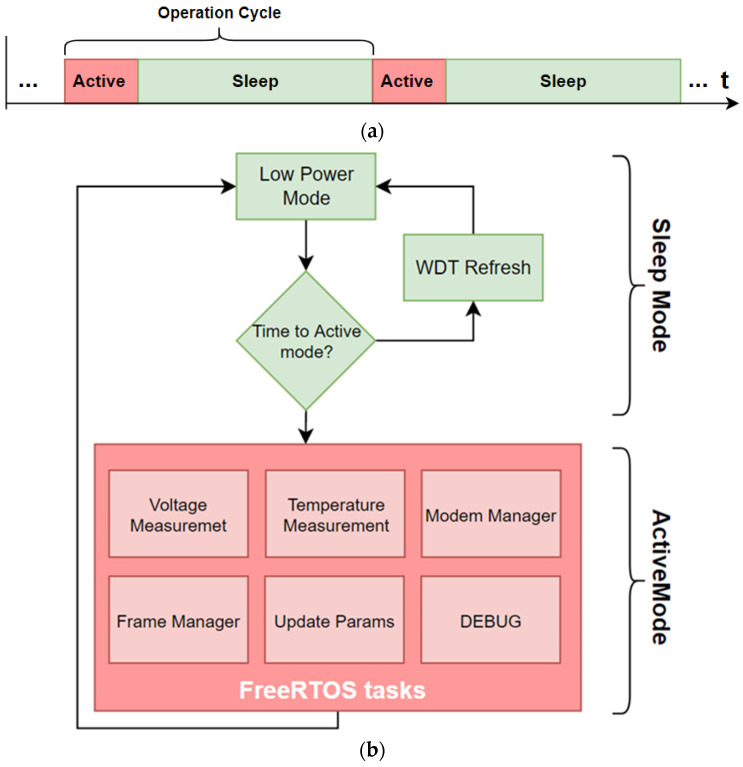
(**a**) Operation cycle scheme. (**b**) Time flow diagram.

As it is detailed in this section, since the nodes perform several tasks that must be performed simultaneously, it is necessary to use an embedded real-time operating system such as FreeRTOS. In addition, the nodes, which monitor four cells and work independently from other nodes, have an operation cycle composed of two operating modes (Figure 5a): active mode, managed by FreeRTOS, where the running tasks are managed and prioritized by the operating system, and ultra-low power mode, which takes most of the duration of operation cycle (more than 95%), where only the temporary synchronization of the node and protection against unknown states are maintained through the WatchDog Timer (WDT).

A flow diagram of the device behavior is shown in Figure 5b. In active mode, the operating system performs tasks such as “Voltage Measurement”, where the diode voltages of the four cells acquired and the temperature of its electrolyte are acquired by the “Temperature Measurement” task, and then are digitized locally in order to make the measurements sent independent of the sensor model used. Subsequently, in the “Frame Manager” task, the resulting data are encoded and grouped into different information packets according to compromise rules between the payload, number of frames, and airtime. In parallel with the “Frame Manager” runs the “Modem Manager” task, which is responsible for the management and control of the LoRaWAN transceiver through a UART interface and AT commands for sending these data frames. Currently, the information is divided into three different frames: the first one indicates the voltage of the four monitored cells; the second sends the supply voltage of the device; and the third sends the electrolyte temperature of the monitored cells.

Moreover, the node can remotely adapt the parameters (such as number of samples, saturation values, sampling time, etc.) involved in the parameterization of the node functionality, providing the devices with the ability to adapt to changing working conditions. In this process, the information received from the central server through the LoRaWAN gateway follows the next task flow: “Modem Manager” and “Update Params”.

After performing the tasks in active mode, the device goes into deep power sleep mode, where only time synchronization via RTC and WDT are active, in order to avoid blocking due to unknown operating states. The reason is that, as previously mentioned, the minimization of energy consumption is one of the main requirements of the design, with the aim of maximizing the lifetime of the devices, especially during the harvesting of anodes and/or cathodes, where the cells are off and therefore the node does not have any external power supply. This operation mode allows for energy saving. The functional flow chart of the sensor nodes is shown in Figure 5b.

Finally, the device has an additional task called “Debug”, which is responsible for informing the user of the status of the tasks in real-time through a UART interface.

### 4.2. Server and Graphical Interface

Once the sensor devices send the collected cell voltage and temperature information to the gateway, the latter resends it to the central server, which processes said info and offers the results to the final users. For short-circuit detection, a multilayer perceptron (MLP) artificial neural network was trained using experimental measurements obtained in the first phase of operation, which were labeled with the existence or not of a short circuit, based on the experience and operations carried out by plant workers. The MLP neural network architecture is defined with eight inputs, four outputs, and two hidden layers of eight neurons each, and they were trained with the Keras package on Tensorflow in Python. Finally, the selected activation functions were Rectified Linear Unit (ReLU) for the hidden layers and Sigmoid for the output layers.

The development of the server software is based on a deployment of microservices on a system of virtual containers managed by Docker. In addition, to guarantee security and access to information, the server offers secure connections (https) and a user and role management module.

**Figure 6:**
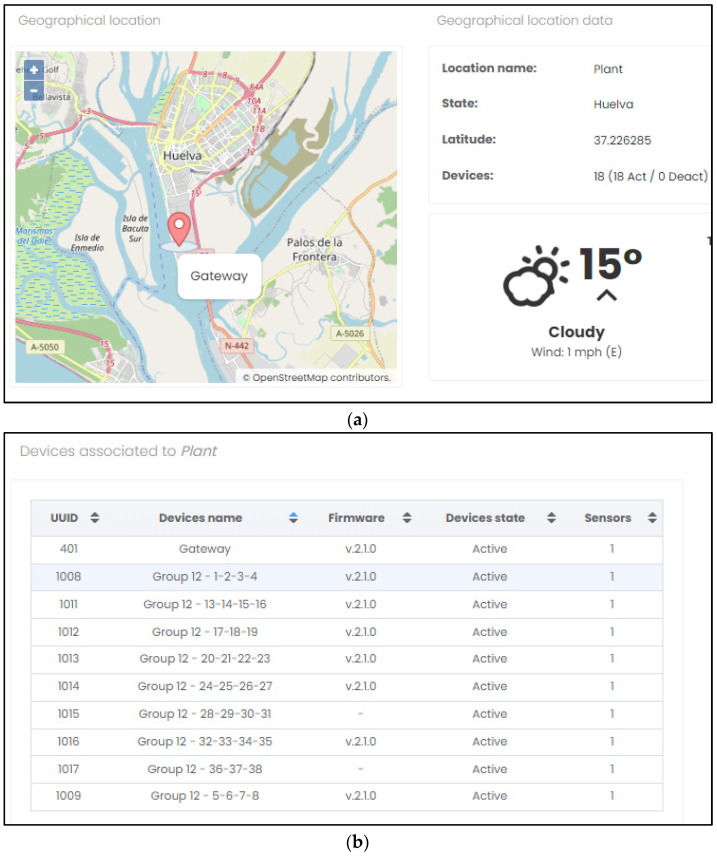
Graphical interface: (**a**) Plant location. (**b**) Detail of deployed nodes.

These microservices are initially responsible for decoding and storing said data in the system’s database (MongoDB). Finally, the data, after being processed by the backend (Node.js), are presented to the user through the VUE framework (Figure 6a,b).

Regarding the services they offer, the server and its graphical user interface were developed for real-time monitoring and detection of short circuits based on cell voltage and temperature data. The interface provides easy access to cell status, performance, system diagnostics, and measurement trends and data. The software application allows the user to visualize the data from each sensor node, both by table or graphically, to find cells with poor status or deviating temperatures. Moreover, in manager mode, the web application also allows the user to reprogram the nodes, adjust internal parameters related to the measurements or the network configuration, and manage the database (export, import, delete, and apply factory settings), as well as user management (create, delete, and modify information and role) and access to the debug section, where the system log is detailed.

## 5. Experimental Results

### 5.1. Laboratory Test

**Figure 7:**
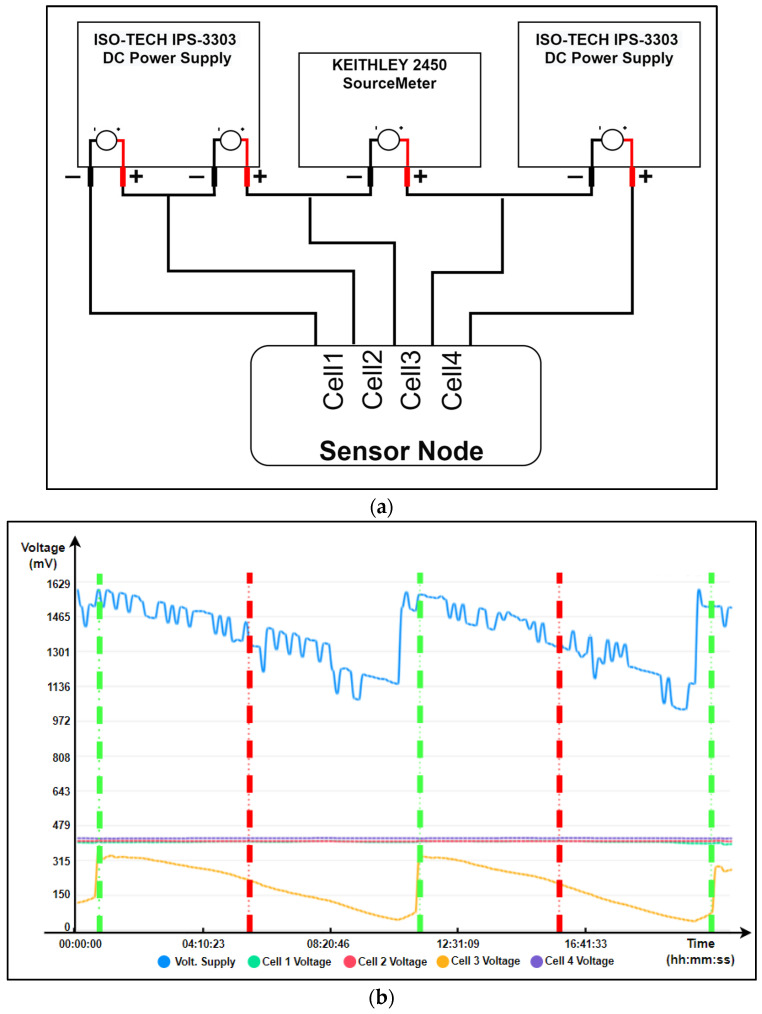
Laboratory test (**a**) Setup. (**b**) Results.

Before deployment for real environment tests, the system validation was carried out in a battery of laboratory tests. For this reason, the behavior of a four-cell group, with three of them at a constant voltage and the other one with a decreasing temporal dynamic, was emulated. This last characteristic represents the emulation of the process of generating a short circuit in an electrolytic cell, and it has been implemented using three channels of two ISO-TECH IPS-3303 continuous power supplies and one programmable channel of a Keithley 2450 Supply, which were configured as can be seen in Figure 7a, and whose results were sent from the end device to the graphical interface.

As seen in Figure 7b, both the voltages of each cell and the input to the source of the sensor device are shown; it also can be seen how the voltage of channels 1, 2, and 4 remains constant at 400 mV DC, while the power supply is between 1.6 V and 1.1 V. On the other hand, channel 3 shows a periodic cycle of 12 h, where the evolution of the voltage is similar to the process of generating a short circuit in a cell (decreasing voltage, from 350 mV to 50 mV with respect to the rest of the channels) and its removal by an operator (recovery of the original voltage).

The vertical lines represent the moments when the node detects the existence (red) or not (green) of a short circuit in cell 3. As can be seen, the detection time of a short circuit since it begins to be generated is approximately 4 h.

At this point, once the behavior of all the elements of the system has been validated, the next step is to install the devices in the field and validate their operation, as detailed in the following section.

### 5.2. Field Test

**Figure 8:**
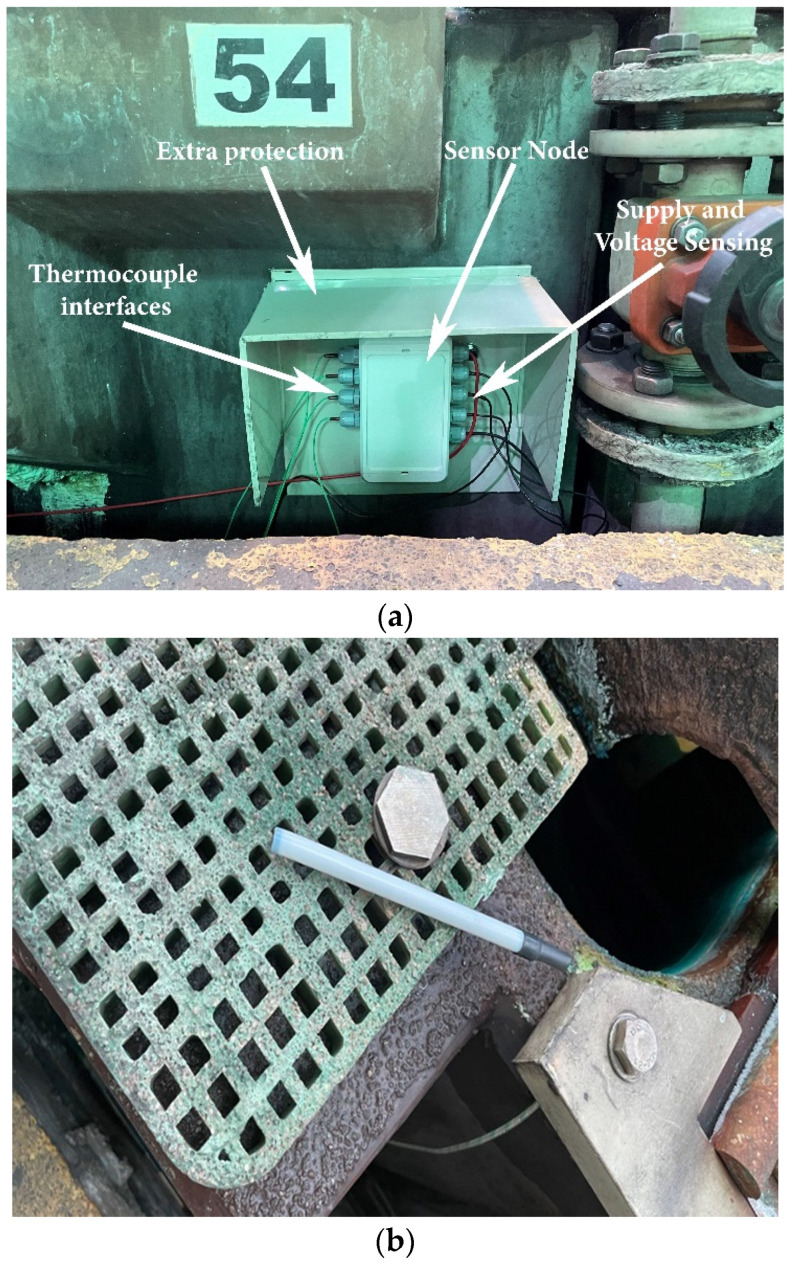
(**a**) Sensor node installed to cells at the Atlantic Copper factory (Huelva, Spain). (**b**) Thermocouple with protection capsule.

The wireless monitoring and control system was deployed in the Atlantic Copper company in Huelva, southwest Spain, which has five decades of experience in the chemical industry. The industrial environment is aggressive for electronics due to high corrosion levels, moderate temperature, large magnetic fields, and liquid spills. In order to test the system, operators installed the sensor–actuator nodes in a semi-group of electrolytic cells. The sensor node was mounted on a profiled backing plate. The plate was used for attaching the node to the electrolytic cell with a simple mounting mechanism, providing additional protection for electrolyte spills, as shown in Figure 8a. As it was said previously, each sensor node managed four adjacent cells, thus simplifying the infrastructure, and measured their low voltage and electrolyte temperature. The measured cell voltages were also used to power the node. The connection to the cell bus bar was made with short wires, and thermocouples with the protection capsule (Figure 8b) were placed inside each cell and connected to the node.

**Figure 9:**
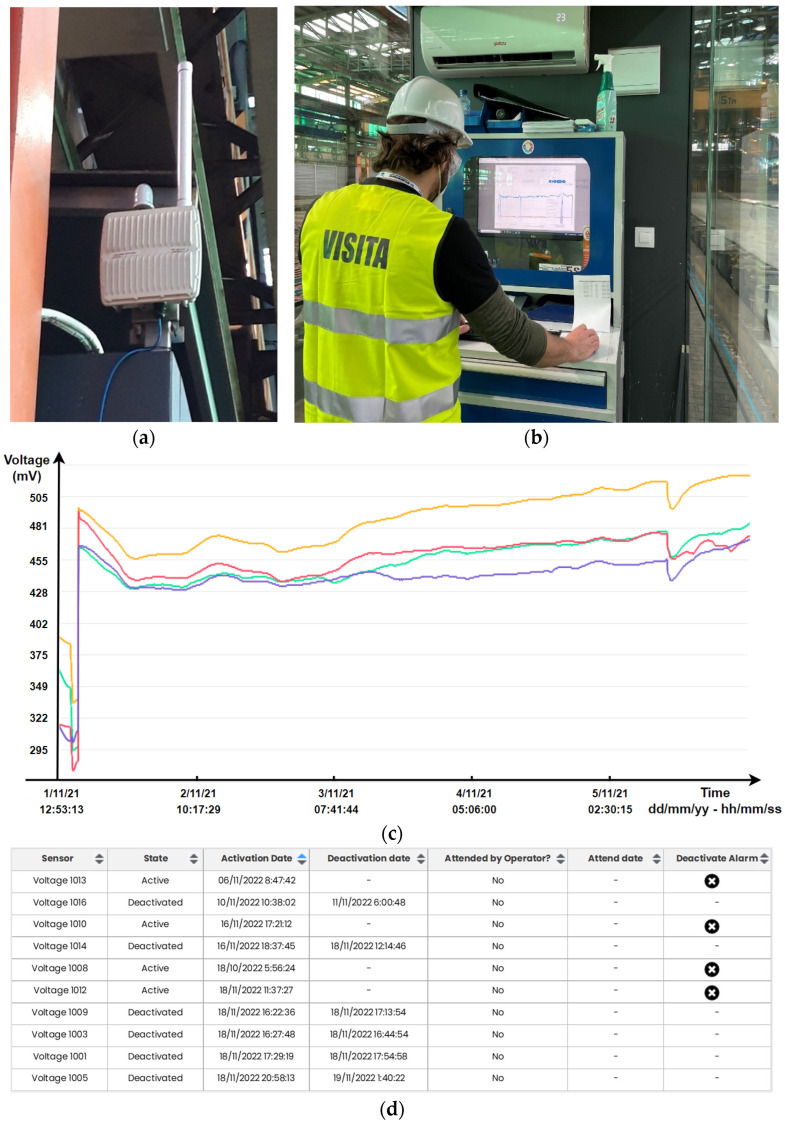
(**a**) Deployed gateway (**b**) and server. (**c**) Graphical interface of four-cell voltage evolution (**d**) and short-circuit alarm management graphical panel.

In addition to the 17 sensor devices deployed, the LoRaWAN gateway (Figure 9a) was installed at the center of the operations plant, where it was previously verified that the level of coverage generated by it in the rest of the plant was enough to guarantee wireless transmissions from the sensor nodes. Finally, both the system server and the screen that offers its graphical interface (Figure 9b) were deployed next to the gateway, since this is the most accessible point in the plant, which facilitated the system’s use by the operators and, therefore, maximized its use.

Once the entire system was deployed and the electrolysis process began in the cells, the nodes were cell-powered at 1.2 Vdc, started up, and connected to the LoRaWAN network automatically, thus beginning their cycle of operation on the 68 monitored cells. This happened until the moment of pulling the anodes and/or cathodes (harvest stage), when the electrolysis stopped, as shown in Figure 9c and, therefore, the nodes lost their power supply. This is where the sensor device nodes showed their condition of very low energy consumption, achieving the transmission of data up to three operation cycles autonomously.

After the first cycles of operation, a remote calibration of the sensor nodes was performed from the server, after which the exploitation phase began (Figure 9c).

In order to quantify the performance of the system, a comparison between the actions recorded by the plant operators without taking into account the sensor devices and the data offered by the sensor devices themselves (Figure 9c,d), which were sent to the server with 98% success, was performed.

Regarding the detection of short circuits, during a testing period of 1 month and 17 days, the operators, who followed the visual and traditional methodology, detected 67% of the total short circuits that happened (61), while the sensor devices detected 97% of them. Moreover, these events were predicted by the sensors (with respect to the moment of detection by the operators) in an average time of 10.5 h, reaching a peak of 32 h, demonstrating the accuracy of the system and its ability to predict short circuits, maximizing, therefore, the performance of the plant. In addition, the developed system detected the non-deactivation of the short circuits after the action of the operators in 20% of the cases, which also places this work as an element of assistance to the operators.

## 6. Conclusions

The formation of short circuits between anode and cathode is a major problem in copper refineries. They reduce electrical efficiency, cause degradation of cathode quality, and increase scrap rate and labor demand. This work tackled such challenges by designing an industrial monitoring system based on harvester-powered intelligent and unattended wireless devices that collaboratively predict impending electrolytic cell failures and perform real-time fault diagnosis. For that purpose, a low-cost sensor device composed by a core and a sensor board with LoRaWAN communication, which guarantees a reliable communication in an industrial environment, was designed and integrated with a remote cloud server following an IoT architecture.

Thus, the operators receive reliable and real-time information on the electrolytic cell performance and state, as demonstrated in the experimental results section. The laboratory and real field tests showed how the designed system can detect up to 30% more of the short circuits currently detected by the team of plant workers, reaching 97%, with an average prediction time of 10.5 h over the detection time by the operators, reaching 32.5 h of maximum prediction time. Therefore, these results enable improvements in operational and energy efficiency, operator safety, process maintenance, and more consistent and higher cathode copper quality.

## Data Availability

Not applicable.
